# Measurement Invariance and Differential Item Functioning of the Health Literacy Instrument for Adults (HELIA): A Large-Scale Cross-Sectional Study in Iran

**DOI:** 10.3390/healthcare10102064

**Published:** 2022-10-17

**Authors:** Mehran Alijanzadeh, Chung-Ying Lin, Rafat Yahaghi, Jalal Rahmani, Nahid Yazdi, Elahe Jafari, Hashem Alijani, Narges Zamani, Razie Fotuhi, Elham Taherkhani, Zeinab Buchali, Robabe Jafari, Narges Mahmoudi, Leila Poorzolfaghar, Safie Ahmadizade, Azam Shahbazkhania, Zainab Alimoradi, Amir H. Pakpour

**Affiliations:** 1 Social Determinants of Health Research Center, Research Institute for Prevention of Non-Communicable Diseases, Qazvin University of Medical Sciences, Qazvin 3419759811, Iran; mehranalijanzadeh@gmail.com (M.A.); yahaghirafat@yahoo.com (R.Y.); jrahmani49@gmail.com (J.R.); n.yazdi@qums.ac.ir (N.Y.); elhejafari@gmail.com (E.J.); hs.alijani@gmail.com (H.A.); zamani.ee@gmail.com (N.Z.); m.r.a.mafi@gmail.com (R.F.); etaherkhani.mid1@gmail.com (E.T.); bochalizeinab@gmail.com (Z.B.); ro.midewife@yahoo.com (R.J.); mahmoudi.narges@yahoo.com (N.M.); poorzolfaghar6565@gmail.com (L.P.); ahmadizadehn@yahoo.com (S.A.); aazam.shahbazkhani@gmali.com (A.S.); zainabalimoradi@yahoo.com (Z.A.); 2 Institute of Allied Health Sciences, College of Medicine, National Cheng Kung University, Tainan 70101, Taiwan; cylin36933@gmail.com; 3 Biostatistics Consulting Center, National Cheng Kung University Hospital, College of Medicine, National Cheng Kung University, Tainan 70101, Taiwan; 4 Department of Nursing, School of Health and Welfare, Jönköping University, 55318 Jönköping, Sweden

**Keywords:** confirmatory factor analysis, factor structure, health literacy, psychometrics, Rasch, validity

## Abstract

Health literacy is important for health behavior engagement. Therefore, it is important to have a good instrument assessing health literacy with a theoretical framework. The present study aimed to examine the measurement invariance and differential item functioning (DIF) of a newly developed health literacy instrument; that is, the Health Literacy Instrument for Adults (HELIA). Confirmatory factor analysis (CFA) and Rasch models were used to examine the data collected from a large Iranian sample (*N* = 9775; 67.3% females; mean age = 36.44 years). All the participants completed the HELIA. CFA was used to examine if the HELIA had a five-factor structure (including reading, access to information, understanding, appraisal, and decision making/behavioral intention factors) and multigroup CFA to examine if the five-factor structure of HELIA was invariant across gender, educational level, accommodation, and age subgroups. Rasch models were used to examine whether each factor of HELIA was unidimensional and DIF contrast in Rasch to examine if the HELIA items were interpreted similarly across the aforementioned subgroups. The CFA results supported the five-factor structure of HELIA, and the Rasch models verified that each HELIA factor is unidimensional. Additionally, multigroup CFA supported the measurement invariance of HELIA across the following subgroups: male vs. female; highly educated vs. poorly educated; city residents vs. suburban residents; and younger age vs. older age. The DIF contrasts in the Rasch models additionally showed that there are no substantial DIF items in the HELIA across aforementioned subgroups. Therefore, the HELIA is a feasible and comprehensive instrument assessing health literacy across different populations in Iran.

## 1. Introduction

Health literacy is an important factor contributing to people’s health behaviors; when people possess good knowledge of health literacy, they engage in more healthy behaviors and less unhealthy behaviors [1,2]. The definition of health literacy is complicated as it involves different components, such as the ability to read, to write, to comprehend, and to understand health information [3]. The World Health Organization (WHO) has defined health literacy as “the cognitive and social skills which determine the motivation and ability of individuals to gain access to, understand and use information in ways which promote and maintain good health” [4]. Assessing health literacy is important because good health literacy can help people develop adaptive coping mechanisms [5], while poor health literacy may initiate people adopting maladaptive coping mechanisms [6]. Specifically, when a person has good health literacy, they can search proper and positive strategies when encountering health problems. In contrast, when a person has poor health literacy, they may not find appropriate methods to cope with health problems. Therefore, when healthcare providers or relevant stakeholders want to assess people’s health literacy, several aspects associated with health literacy should be simultaneously considered.

The current literature has thus developed different types of instruments assessing health literacy to satisfy the needs of assessing health literacy across different populations. To date, the most used instruments assessing health literacy include the Newest Vital Sign (NVS) [7], the Test of Functional Health Literacy in Adults (TOFHLA) [8], and the Rapid Estimate of Adult Literacy in Medicine (REALM) [9]. Although these instruments have covered some components of health literacy, prior research has concluded that these instruments do not have comprehensively conceptual dimensions to thoroughly assess health literacy [10]. Additionally, these instruments were developed only for a clinical setting perspective and might not cover a wide range of populations, such as the general population [11]. Therefore, it is important to reassess the instruments’ psychometric properties to accumulate scientific evidence, even if the instrument has been in use for a long time [12].

The current trend in health literacy instruments focuses on general health literacy assessment because it can be used for a wide range of purposes. Specifically, three types of health literacy instruments have been proposed and developed: (i) general instruments, (ii) condition-specific (disease or content) instruments, and (iii) instruments of specific populations (e.g., older people or children) [10]. Although condition-specific instruments and instruments for specific populations have the advantages of high sensitivity for specific populations, they are lacking for group comparisons. In contrast, general instruments can be used for group comparisons. Therefore, HELIA could be a potential health literacy instrument to understand the levels of health literacy across different populations.

Apart from the three instruments mentioned above, Haun et al. [10] reviewed 51 health literacy instruments and summarized that the literature needs a new measure assessing health literacy, taking into consideration the use of a theoretical framework. Accordingly, some health literacy instruments have been developed, including the Health Literacy Questionnaire (HLQ) [13], the European Health Literacy Survey Questionnaire (HLS-EU-Q) [14], and the Health Literacy Instrument for Adults (HELIA) [15]. The HLQ and HLS-EU-Q were developed from the Western populations, while the HELIA was developed using the lens of a non-Western culture. Nevertheless, it is suggested that a good instrument assessing health literacy should have the features of (i) applicability to different populations, (ii) a wide range of domains corresponding to a good theoretical framework, and (iii) good psychometric properties ensuring consistent results in evaluations [16]. Therefore, the present study aimed to add value to the current literature via validating a health literacy instrument developed with a rigorous theoretical background in an Eastern culture [17]. It is important to ensure health literacy instruments developed from the lens of Eastern cultures as well because Western and Eastern cultures may possess different viewpoints toward health, especially in terms of the Western context being individualistic and the Eastern being collectivistic. For example, Western people consider health a personal matter; therefore, they may develop their health literacy based on professionals. In contrast, Eastern people based on collectivism may share health information among relatives and friends more frequently.

The HELIA was initially developed from a group of experts in Iran [15], originally named Health Literacy for Iranian Adults and later renamed the Health Literacy Instrument for Adults [11] to broaden the potential population from Iranians to the worldwide population. Although the full names are different, the abbreviated names are the same: HELIA. The content validity of the HELIA was first established by Montazeri et al. [15], and later, Tavousi et al. [11] extended the HELIA’s psychometric properties to the following aspects: (i) item deletion to shorten the HELIA, retaining 33 items with satisfactory factor loadings in the exploratory factor analysis (EFA); (ii) a five-factor structure using the retained 33 items concluded by the EFA; (iii) the five-factor structure confirmed by the confirmatory factor analysis (CFA); and (iv) satisfactory internal consistency for the five factors in the shortened HELIA, as well as the full HELIA. The five factors found in the HELIA are reading, access to information, understanding, appraisal, and decision making/behavioral intention.

Although the HELIA has been validated, the present study observed a gap in that the psychometric properties of HELIA need to be better understood. Specifically, the HELIA has never been examined for its measurement invariance [18] or differential item functioning (DIF) [19]. Putnick and Bornstein [18] proposed the need and importance of testing measurement invariance in psychological research. Therefore, establishing evidence regarding measurement invariance via CFA and DIF using Rasch models for HELIA is essential because such evidence supports a fair comparison across groups with different features (e.g., male vs. female or highly educated vs. poorly educated). With such a fair comparison, health literacy information can be correctly assessed and compared across these groups. As a result, additional psychometric evidence for the HELIA is needed, and this echoes the nature of science that the literature needs cumulated psychometric evidence to repeatedly examine the robustness of any developed instrument [20,21,22,23]. The present study aimed to use a large sample from Iran to reexamine the factor structure of the HELIA using two advanced psychometric testing methods (i.e., CFA and Rasch analysis). Moreover, measurement invariance and DIF were assessed for the HELIA across the following subgroups: male vs. female; highly educated vs. poorly educated; city resident vs. rural resident; and younger age vs. older age.

## 2. Materials and Methods

### 2.1. Study Design and Participant Recruitment Procedure

This cross-sectional study was conducted in Qazvin (a province 150 km northwest of Tehran). A multistage stratified cluster sampling approach was used to collect data. Qazvin province was stratified into 70 strata, and several health centers were randomly selected from each stratum based on the size of the population. Although the health centers are located in different cities in Qazvin province, the residents shared the same spoken language (i.e., Persian). From each health center, families were then randomly selected from the list of the families in that health center, adopting a similar recruitment procedure to that of other studies [24,25]. The same recruitment procedure was used because (i) this recruitment procedure was the most precise in sampling the population from Qazvin province and (ii) the present study had similar sources to the previous two studies [21,22], with full collaboration from all health departments in Qazvin province, including remote rural areas. Twenty-five trained research associates contacted the families to ask them to participate in the study. Upon their agreement, the participants were asked to complete a form containing the study aims and questionnaires. Specifically, the same 25 research associates administered the questionnaires by paper and pencil. Given that we aimed to select study participants from urban cities and big rural areas, collecting data from rural and cities areas and the size of the population were the most important demographic factors for sampling. That is, we have to know the population size of each clustered area to decide how many participants should be recruited for each clustered area. For example, there were 43,798 residents in the area in Avaj City, and we have to recruit 330 participants for the area in Avaj City. The participants’ response rate was 69%. All procedures conducted were approved by the Ethics Committee of Qazvin University of Medical Sciences (IR.QUMS.REC.1400.225). Written Informed consent was obtained from all study participants.

### 2.2. Measures

#### 2.2.1. Health Literacy Instrument for Adults (HELIA)

The HELIA contains 33 items rated on a five-point Likert scale with the following response choices: never (score 1), rarely (score 2), sometimes (score 3), usually (score 4), and always (score 5). A lower score indicates a lower level of health literacy for the respondents. The 33 items distributed into five factors: reading (four items), access to information (six items), understanding (seven items), appraisal (four items), and decision making/behavioral intention (12 items). Prior psychometric evidence of the HELIA shows that it is a psychometrically sound instrument assessing health literacy for Iranians [11,15].

#### 2.2.2. Demographic Information

The present participants self-reported the following demographic information: age (in years), gender (male or female), residency (Qazvin, Takestan, Avaj, Abyek, Bueenzahra, or Alborz city), educational level (primary school, secondary school, diploma, or university), accommodation (city or rural), and marital status (single, married, or divorced/widowed).

### 2.3. Data Analysis

The sample’s characteristics were analyzed using the frequency (%) and mean (SD). Afterward, two methods of psychometric testing (i.e., classical test theory and Rasch models) were used to examine the HELIA. In the classical test theory, confirmatory factor analysis (CFA) with a diagonally weighted least squares (DWLS) estimator was used to fit the five-factor structure of the HELIA (i.e., reading, access to information, understanding, appraisal, and decision making/behavioral intention). Moreover, internal consistency (via Cronbach’s α and McDonald’s ω), together with the item–total correlation, were calculated. The skewness and kurtosis of every HELIA item score were checked. Composite reliability and average variance extracted were computed using the factor loadings derived from the CFA findings. In the CFA, fit indices of a comparative fit index (CFI) > 0.9, a Tucker–Lewis index (TLI) > 0.9, a root mean square error of approximation (RMSEA) < 0.08, and a standardized root mean square residual (SRMR) < 0.08 were used to indicate satisfactory data–model fit [26,27]. Moreover, α and ω > 0.7 indicates good internal consistency [28]; composite reliability >0.7 and average variance extracted >0.5 indicate that the HELIA items capture good percentages of variance in the HELIA factors [29].

In the Rasch models, a partial credit model was used for the estimation in each HELIA domain. Difficulty and discrimination coefficients were calculated. Two types of mean square (MnSq), infit and outfit, were used to examine if every HELIA item fits in its embedded domain. Differential item functioning (DIF) was employed for the HELIA items across the following demographic variables: gender (male vs. female), educational level (lower than diploma vs. higher than diploma), accommodation (city vs. rural), and age (below mean age (i.e., 36.44 years) vs. above mean age). Separation reliability and separation index (including item separation and person separation) were also calculated. For the infit and outfit MnSq, values between 0.5 and 1.5 indicate good fit [30]. For DIF, a DIF contrast less than 1 indicates no substantial DIF [31,32]. For separation reliability, a value > 0.7 indicates acceptability [33]. For the separation index, a value > 2 indicates adequateness [34].

All statistics were analyzed using JASP (version 0.16.3), except for the Rasch models, which were established using WINSTEPS.

## 3. Results

This large-scale study included 9775 participants (3073 males; 31.4%) residing across Qazvin province in Iran, with most living in Qazvin city (*n* = 4655; 47.6%), Takestan city (*n* = 1216; 12.4%), and Alborz city (*n* = 1867; 19.1%). On average, the participants were aged 36.44 ± 11.97 years. Moreover, the present sample was generally well-educated (over 70% had a diploma or a degree) and living in a city (*n* = 7287; 74.6%). Table 1 reports the detailed information of the present sample’s demographics.

Regarding the item properties of the HELIA in the classical test theory (Table 2), they showed strong factor loadings (0.795–0.921 for the reading factor; 0.822–0.886 for the access to information factor; 0.688–0.839 for the understanding factor; 0.782–0.906 for the appraisal factor; and 0.682–0.787 for the decision making/behavioral intention factor), satisfactory item-to-total correlation (0.724–0.816 for the reading factor; 0.723–0.824 for the access to information factor; 0.694–0.795 for the understanding factor; 0.666–0.782 for the appraisal factor; and 0.620–0.741 for the decision making/behavioral intention factor), and relatively normal distribution (skewness = 0.682–0.921 for all items; kurtosis = −0.969 to 1.804 for all items). In addition to the good properties at the item level, the HELIA showed good properties at the scale level (Table 3). More specifically, all of the HELIA domains had good fit in the CFA five-factor structure (CFI = 0.993, TLI = 0.993, RMSEA = 0.033, and SRMR = 0.041), excellent composite reliability (0.903–0.942), adequate average variance extracted (0.555–0.753), and satisfactory internal consistency (α = 0.876–0.926; ω = 0.879–0.926).

**Figure 1:**
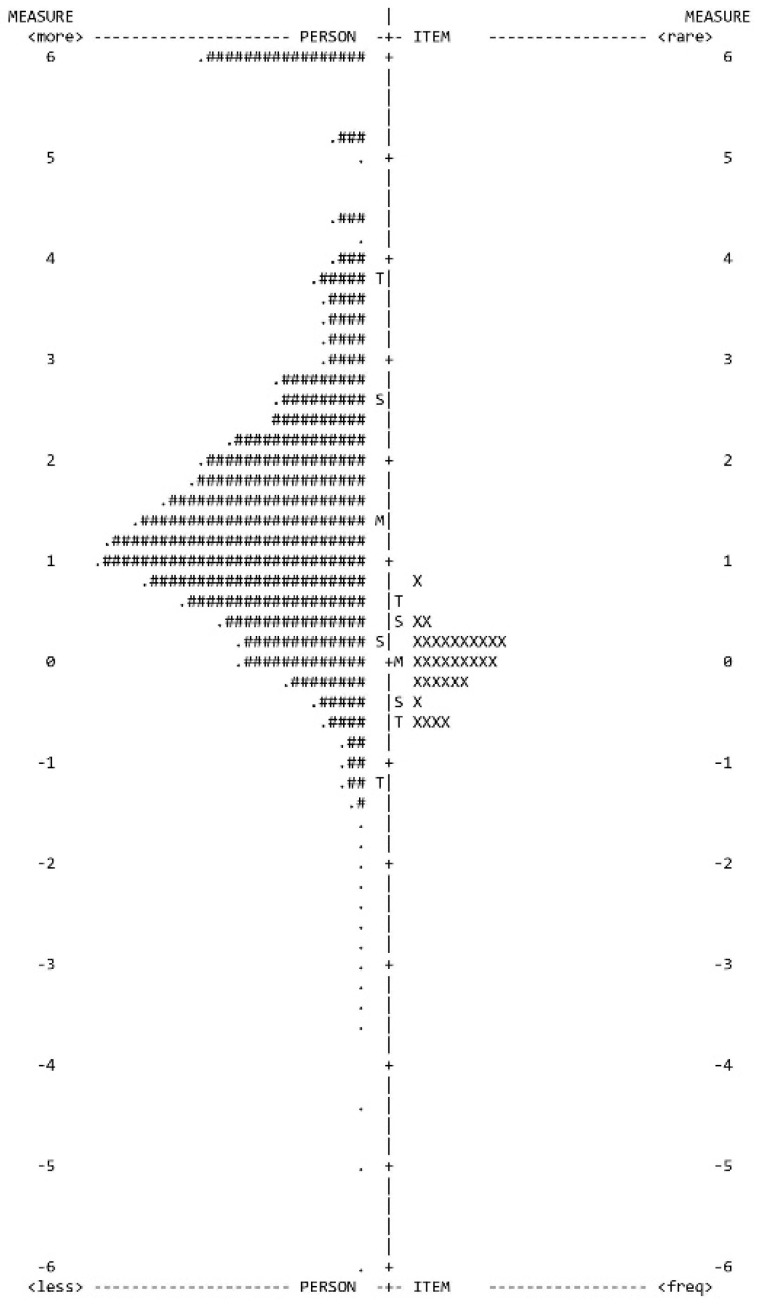
Wright map for the HELIA. RE = reading; ATL = access to information; UND = understanding; APP = appraisal; DM = decision making/behavioral intention. Each # indicates 61 participants.

The Rasch principal component analysis results showed that the HELIA is multidimensional, as the eigenvalue of the unexplained variance in the first contrast was 4.68, in the second contrast was 2.64, in the third contrast was 2.35, and in the fourth contrast was 2.03. Regarding the item properties of the HELIA in the Rasch models (Table 2), they showed a wide range of difficulty coefficients (−0.38 to 0.29 for the reading factor; −0.51 to 0.50 for the access to information factor; −0.52 to 0.76 for the understanding factor; −0.50 to 0.33 for the appraisal factor; and −0.62 to 0.79 for the decision making/behavioral intention factor), relatively stable discrimination coefficients (0.68–1.26 for all items), and acceptable infit and outfit MnSq (infit MnSq = 0.74–1.36 for all items; outfit MnSq = 0.75–1.32 for all items). A Wright map of the HELIA is presented in Figure 1 to show the item coverage, the item–person targeting, and the items’ conundrum. Moreover, no substantial DIF items were observed across gender (DIF contrast = −0.22 to 0.23), educational level (DIF contrast = −0.28 to 0.56), accommodation (DIF contrast = −0.20 to 0.15), or age group (DIF contrast = −0.35 to 0.36). Additional tests on the DIF across these cities also showed no substantial DIF (DIF contrast = 0.93 to −0.79). Moreover, local independence was supported by the residual correlations of less than 0.5.

The scale level of the HELIA also had good properties in the Rasch analysis results: good separation reliability (item separation reliability = 0.99–1.00; person separation reliability = 0.78–0.85) and an acceptable separation index (item separation index = 11.81–24.03; person separation index = 1.86–2.34).

## 4. Discussion

The present study reexamined the structure of the HELIA factors with the use of a large and nearly representative sample in Qazvin, Iran. Specifically, we examined the psychometric properties, measurement invariance, and DIF information of the HELIA. Our findings bridge the gap in the literature and show that the HELIA is a psychometrically sound and measurement invariant instrument assessing health literacy among Iranians. The findings indicate that the HELIA contains a five-factor structure covering a wide spectrum of the health literacy concept [3,4,11]. Moreover, the construct validity of the HELIA was verified using two different psychometric testing methods: the CFA results found that the full HELIA has a five-factor structure with satisfactory loadings for each item; the Rasch results found that all of the factors in the HELIA are unidimensional. Additionally, the measurement invariance and DIF of the HELIA were supported by the present study’s findings. Specifically, the entire five-factor structure was found to be invariant across the following subgroups: male vs. female; poorly educated vs. highly educated; city resident vs. suburban resident; and younger age vs. older age. No substantial DIF items were observed among the aforementioned subgroups. Although good properties of the HELIA were found in the present study, caution should be taken for scoring of the HELIA. Specifically, the HELIA cannot be used as a total score and should rather be used as a separate dimension score (e.g., reading subscale score). Specifically, the Rasch principal component analysis results indicated that the HELIA is multidimensional; therefore, the full HELIA cannot be used as a total score.

The present findings align with prior evidence on the HELIA [11] regarding the five-factor structure. Therefore, the five factors of reading, access to information, understanding, appraisal, and decision making/behavioral intention are clear constructs that should be adopted by healthcare providers. Using the five factors, healthcare providers could assess people’s (especially Iranians’) health literacy in specific constructs. Then, different approaches or foci can be used to improve health literacy. For example, if a person is found to have low scores in the reading subscale of the HELIA, healthcare providers can help improve their reading ability to obtain health information or healthcare providers can find alternative ways other than reading to help the person to obtain health information. Moreover, the five-factor structure satisfies the multidimensional requirements proposed by prior experts and committees [4,14].

Although the development process of the HELIA fulfills the scientific rigor (for detailed information, please refer to Tavousi et al. [11] or Montazeri et al. [15]), its development procedure is different from another two health literacy instruments (i.e., the HLQ developed by Osborne et al. [13] and the HLS-EU-Q by Sørensen et al. [14]). Specifically, the HLQ was developed using live experiences from individuals and professionals at the beginning. After collecting life experiences, existing theories on health literacy were used and adopted for comparison with the experiences to generate the HLQ items for further psychometric testing [13]. The development stage of the HLS-EU-Q began with using a systematic review on the definition of health literacy that includes different aspects of health literacy. Later, the Delphi procedure and focus groups were used for generating the HLS-EU-Q items [14]. In contrast to other health literacy instruments, the HELIA was developed with a health literacy model proposed by Ratzan and Parker [35]. Accordingly, the constructs of the HELIA are somewhat different from other health literacy instruments (e.g., the HLQ has the following nine domains: feel understood and supported by healthcare providers, have sufficient information to manage my health, actively managing health, have social support for health, critically appraise health information, ability to actively engage with healthcare providers, navigating the healthcare system, ability to find good health information, and ability to understand health information well enough to know what to do; meanwhile, the HLS-EU-Q has the following three domains: accessing, understanding, and appraising and applying). Later, strong research and expert committee reviews were applied for item generation. Therefore, the HELIA can also be viewed as a theoretically driven instrument assessing health literacy.

In addition to confirming the factor structure, the present findings indicate that the factor structure is invariant and that the HELIA items are comparable across a variety of subgroups without serious bias in interpretation. People with different features (e.g., gender, educational level, living environment, and age) may have different experiences and thoughts in interpreting item descriptions, and such differences may result in inappropriate comparisons between groups [15,18]. Moreover, the HELIA was found to be invariant across city residency in the present sample, which implies that the HELIA can be used for the entire Qazvin province in Iran. Therefore, it is important for healthcare providers to ensure the measurement invariance and DIF for an instrument before using it to assess a construct. Subsequently, the supported measurement-invariant and DIF-free HELIA is a robust instrument assessing health literacy across groups with different features. With the measurement invariance and DIF-free evidence found for the HELIA, healthcare providers and researchers can use the HELIA to obtain unbiased health literacy information across different subgroups [18]. This is especially important for the present invariance findings for different levels of education. Specifically, educational level is a key factor contributing to health literacy, and the invariance finding in educational level ensures that the HELIA can be used for people with different levels of education from primary school to university.

There are some limitations in the present study. First, the present study did not use other external criterion measures to examine if the HELIA has good concurrent validity with associated constructs. Therefore, future studies may want to extend the present study’s findings to explore if factors associated with health literacy have adequate associations with the HELIA and its domains. Second, the present study did not assess test–retests of the HELIA, and the reproducibility of the HELIA remains unclear. Third, an important psychometric feature of responsiveness was not examined. Therefore, it is unclear if the HELIA can be used to evaluate program effectiveness in health literacy improvements. Future studies are thus required to investigate the responsiveness of the HELIA to extend its usefulness in program evaluations. Lastly, although the present study recruited a large sample with relatively good representativeness of Qazvin residents, the present findings cannot be generalized to other Iranian populations (i.e., residing somewhere other than Qazvin province).

## 5. Conclusions

In conclusion, the HELIA is a feasible and comprehensive instrument assessing health literacy across different populations. The HELIA is a general instrument that can be used in different populations and to make group comparisons. However, given the nature of a general instrument assessing health literacy, the HELIA is unable to provide information regarding if target participants possess specific health literacy knowledge. Moreover, future studies are needed to examine if the HELIA has good properties such as responsiveness to ensure its usefulness in evaluating health literacy programs. Nevertheless, the present study findings support the use of the HELIA by healthcare providers and policymakers to understand people’s level of health literacy.

## Figures and Tables

**Table 1:** Participant characteristics (*N* = 9775).

	Mean ± SD or *n* (%)
**Age (years)**	36.44 ± 11.97
**Gender**	
Male	3073 (31.4)
Female	6576 (67.3)
Missing	126 (1.3)
**City**	
Qazvin	4655 (47.6)
Takestan	1216 (12.4)
Avaj	330 (3.4)
Abyek	657 (6.7)
Bueenzahra	963 (9.9)
Alborz	1867 (19.1)
Missing	87 (0.9)
**Educational status**	
Primary school	1082 (11.1)
Secondary school	1576 (16.1)
Diploma	3220 (32.9)
University	3851 (39.4)
Missing	46 (0.5)
**Accommodation**	
City	7287 (74.6)
Rural	2309 (23.6)
Missing	179 (1.8)
**Marital status**	
Single	1779 (18.2)
Married	6987 (71.5)
Divorced/widowed	90 (0.9)
Missing	919 (9.4)

**Table 2:** Psychometric properties of the Health Literacy Instrument for Adults at the item level.

Item #	Analyses from Classical Test Theory				Rasch Analyses							
	Factor Loading *^,†^	Item–Total Correlation	S	K	Infit MnSq	Outfit MnSq	Difficulty	Discrimination	DIF Contrast across Gender ^§^^,¶^	DIF Contrast across Education ^§^^,#^	DIF Contrast across Accommodation ^§^^,#^	DIF Contrast across Age ^§^^,#^
Re-1	0.795	0.724	0.795	0.375	1.16	1.18	−0.38	0. 79	0.10	−0.23	0.11	0.00
Re-2	0.846	0.788	0.846	0.106	0.89	0.89	0.05	1.12	−0.22	0.00	−0.05	0.16
Re-3	0.903	0.816	0.903	0.079	0.81	0.80	0.04	1.21	0.00	0.08	0.10	−0.18
Re-4	0.921	0.753	0.921	−0.110	1.10	1.10	0.29	0.91	−0.10	0.17	−0.15	0.00
AC-1	0.833	0.780	0.833	−0.058	1.00	1.03	−0.01	0.97	0.13	0.16	0.05	−0.14
AC-2	0.850	0.803	0.850	0.096	0.88	0.86	−0.31	1.13	−0.10	−0.14	0.15	−0.18
AC-3	0.886	0.810	0.886	−0.441	0.91	0.92	0.50	1.09	−0.04	−0.11	0.00	−0.07
AC-4	0.879	0.824	0.879	−0.167	0.83	0.82	0.13	1.19	−0.09	−0.08	0.00	−0.09
AC-5	0.859	0.794	0.859	−0.134	0.99	0.99	0.20	1.02	−0.10	0.02	−0.10	0.36
AC-6	0.822	0.723	0.822	0.323	1.36	1.32	−0.51	0.65	0.23	0.16	−0.15	0.12
Un-1	0.700	0.719	0.700	1.283	1.05	1.11	−0.31	0.91	−0.07	−0.16	0.06	0.06
Un-2	0.688	0.758	0.688	1.423	0.93	0.90	−0.40	1.10	−0.10	−0.10	0.04	0.05
Und-3	0.811	0.760	0.811	0.373	0.98	0.99	0.36	1.03	0.10	0.14	−0.11	0.00
Un-4	0.770	0.795	0.770	1.060	0.83	0.84	−0.05	1.18	0.19	0.11	0.00	−0.05
Un-5	0.706	0.768	0.706	1.804	0.96	0.90	−0.52	1.09	−0.07	0.00	0.15	−0.13
Un-6	0.774	0.759	0.774	0.547	0.93	0.97	0.17	1.05	−0.02	−0.28	0.08	0.00
Un-7	0.839	0.694	0.839	−0.003	1.28	1.30	0.76	0.70	0.00	0.24	−0.20	0.04
Ap-1	0.906	0.726	0.906	−0.177	1.11	1.09	0.33	0.92	0.00	0.56	−0.15	−0.35
Ap-2	0.847	0.782	0.847	−0.059	0.81	0.80	0.17	1.21	0.10	−0.10	0.00	0.20
Ap-3	0.810	0.777	0.810	0.009	0.81	0.82	0.00	1.18	0.06	−0.23	0.04	0.12
Ap-4	0.782	0.666	0.782	0.424	1.22	1.25	−0.50	0.72	−0.18	−0.26	0.04	0.05
De-1	0.766	0.644	0.766	0.327	0.94	1.06	−0.25	0.98	−0.07	0.17	0.08	−0.35
De-2	0.689	0.660	0.689	1.007	0.97	0.97	−0.62	1.04	−0.20	0.10	0.00	0.00
De-3	0.714	0.662	0.714	0.084	1.04	1.11	−0.12	0.96	−0.08	−0.05	0.00	0.05
De-4	0.774	0.632	0.774	−0.429	1.30	1.31	0.16	0.72	0.04	0.00	0.00	−0.06
De-5	0.743	0.741	0.743	−0.110	0.77	0.79	−0.03	1.25	−0.07	−0.12	0.09	0.07
De-6	0.766	0.631	0.766	−0.969	1.26	1.29	0.79	0.68	0.13	−0.03	−0.18	0.13
De-7	0.732	0.732	0.732	0.019	0.74	0.75	−0.12	1.26	0.07	−0.12	0.14	−0.12
De-8	0.757	0.717	0.757	0.142	0.82	0.81	−0.23	1.20	−0.13	−0.10	0.14	−0.13
De-9	0.787	0.694	0.787	−0.294	0.99	1.0	0.22	1.03	0.00	0.12	−0.16	0.00
De-10	0.731	0.696	0.731	−0.349	0.92	0.97	0.33	1.06	0.00	−0.10	0.04	0.06
De-11	0.682	0.620	0.682	0.403	1.21	1.24	−0.32	0.81	0.22	0.22	−0.19	0.28
De-12	0.786	0.710	0.786	−0.244	0.94	0.96	0.18	1.08	0.00	−0.03	0.03	0.00

* All factor loadings were significant at 0.001. S = skewness; K = kurtosis; Re = reading; Ac = access to information; Un = understanding; Ap = appraisal; De = decision making/behavioral intention. ^†^ Based on first-order confirmatory factor analysis (CFA). CFA model fit: *χ*^2^ (*df*) = 5052.419 (485); comparative fit index = 0.993; Tucker–Lewis index = 0.993; root mean square error of approximation (90% CI) = 0.033 (0.032, 0.034); and standardized root mean square residual = 0.041. ^§^ DIF contrast >1 indicates substantial DIF. ^¶^ DIF contrast across gender = difficulty for females – difficulty for males. ^#^ DIF contrast across education = difficulty for participants with lower education (lower than diploma) – difficulty for participants with higher education (higher than diploma). ^#^ DIF contrast across accommodation = difficulty for participants living in city areas – difficulty for participants living in rural areas. ^#^ DIF contrast across age = difficulty for younger-aged participants (<36.44 years) – difficulty for older-aged participants (≥36.44 years). MnSq = mean square error; DIF = differential item functioning; S = skewness; K = kurtosis.

**Table 3:** Psychometric properties of the Health Literacy Instrument for Adults at the scale level.

Psychometric Testing	Re	Ac	Un	Ap	De
Composite Reliability	0.924	0.942	0.903	0.904	0.937
Average variance extracted	0.753	0.731	0.574	0.701	0.555
Internal consistency (Cronbach’s α)	0.896	0.926	0.917	0.876	0.920
Internal consistency (McDonald’s ω)	0.897	0.926	0.918	0.879	0.921
Item separation reliability from Rasch	0.99	1.0	1.0	1.0	1.0
Item separation index from Rasch	11.81	17.18	22.53	15.78	24.03
Person separation reliability from Rasch	0.80	0.85	0.78	0.78	0.82
Person separation index from Rasch	1.98	2.34	1.86	1.90	2.15

Re = reading; Ac = access to information; Un = understanding; Ap = appraisal; De = decision making/behavioral intention.

## Data Availability

The data presented in this study will be considered for sharing upon reasonable request to the corresponding author.

## References

[1] Bennett I.M., Chen J., Soroui J.S., White S. (2009). The contribution of health literacy to disparities in self-rated health status and preventive health behaviors in older adults. Ann. Fam. Med..

[2] Osborn C.Y., Paasche-Orlow M.K., Bailey S.C., Wolf M.S. (2011). The mechanisms linking health literacy to behavior and health status. Am. J. Health Behav..

[3] Schnitzer A.E., Rosenzweig M., Harris B. (2011). Health literacy: A survey of the issues and solutions. J. Consum. Health Internet.

[4] World Health Organization (1998). Health Promotion Glossary.

[5] Schneider T., Wolgemuth J.R., Bradley-Klug K.L., Bryant C.A., Ferron J.M. (2022). Perceptions of School Life and Academic Success of Adolescents with Asthma. J. Adolesc. Res..

[6] Easton P., Entwistle V.A., Williams B. (2010). Health in the ‘hidden population’ of people with low literacy. A systematic review of the literature. BMC Public Health.

[7] Weiss B.D., Mays M.Z., Martz W., Castro K.M., DeWalt D.A., Pignone M.P., Mockbee J., Hale F.A. (2005). Quick assessment of literacy in primary care: The newest vital sign. Ann. Fam. Med..

[8] Parker R.M., Baker D.W., Williams M.V., Nurss J.R. (1995). The test of functional health literacy in adults: A new instrument for measuring patients’ literacy skills. J. Gen. Intern. Med..

[9] Davis T.C., A Crouch M., Long S.W., Jackson R.H., Bates P., George R.B., E Bairnsfather L. (1991). Rapid assessment of literacy levels of adult primary care patients. Fam. Med..

[10] Haun J.N., Valerio M.A., McCormack L.A., Sørensen K., Paasche-Orlow M.K. (2014). Health literacy measurement: An inventory and descriptive summary of 51 instruments. J. Health Commun..

[11] Tavousi M., Haeri-Mehrizi A., Rakhshani F., Rafiefar S., Soleymanian A., Sarbandi F., Ardestani M., Ghanbari S., Montazeri A. (2020). Development and validation of a short and easy-to-use instrument for measuring health literacy: The Health Literacy Instrument for Adults (HELIA). BMC Public Health.

[12] Lin C.-Y., Tsai C.-S., Fan C.-W., Griffiths M.D., Chang C.-C., Yen C.-F., Pakpour A.H. (2022). Psychometric Evaluation of Three Versions of the UCLA Loneliness Scale (Full, Eight-Item, and Three-Item Versions) in Taiwanese Sexual Minority Men. Int. J. Environ. Res. Public Health.

[13] Osborne R.H., Batterham R.W., Elsworth G.R., Hawkins M., Buchbinder R. (2013). The grounded psychometric development and initial validation of the health literacy questionnaire (HLQ). BMC Public Health.

[14] Sørensen K., van den Broucke S., Pelikan J.M., Fullam J., Doyle G., Slonska Z., Kondilis B., Stoffels V., Osborne R.H., Brand H. (2013). Measuring health literacy in populations: Illuminating the design and development process of the European health literacy survey questionnaire (HLS-EU-Q). BMC Public Health.

[15] Montazeri A., Tavousi M., Rakhshani F., Azin S.A., Jahangiri K., Ebadi M., Naderimagham S., Solimanian A., Sarbandi F., Motamedi A. (2014). Health Literacy for Iranian Adults (HELIA): Development and psychometric properties. Payesh.

[16] Rowlands G., Trezona A., Russell S., Lopatina M., Pelikan J., Paasche-Orlow M., Drapkina O., Kontsevaya A., Sørensen K. (2019). What Is the Evidence on the Methods, Frameworks and Indicators Used to Evaluate Health Literacy Policies, Programmes and Interventions at the Regional, National and Organizational Levels?.

[17] Shaw S.J., Huebner C., Armin J., Orzech K., Vivian J. (2009). The Role of Culture in Health Literacy and Chronic Disease Screening and Management. J. Immigr. Minor. Health.

[18] Putnick D., Bornstein M.H. (2016). Measurement invariance conventions and reporting: The state of the art and future directions for psychological research. Dev. Rev..

[19] Wang W.-C. (2008). Assessment of differential item functioning. J. Appl. Meas..

[20] Lin C.-Y., Hwang J.-S., Wang W.-C., Lai W.-W., Su W.-C., Wu T.-Y., Yao G., Wang J.-D. (2019). Psychometric evaluation of the WHOQOL-BREF, Taiwan version, across five kinds of Taiwanese cancer survivors: Rasch analysis and confirmatory factor analysis. J. Formos. Med. Assoc..

[21] Tadakamadla S.K., Quadri M.F.A., Pakpour A.H., Zailai A.M., Sayed M.E., Mashyakhy M., Inamdar A.S., Tadakamadla J. (2014). Reliability and validity of Arabic rapid estimate of adult literacy in dentistry (AREALD-30) in Saudi Arabia. BMC Oral Health.

[22] Lin C.-Y., Broström A., Griffiths M.D., Pakpour A.H. (2020). Psychometric evaluation of the Persian eHealth Literacy Scale (eHEALS) among elder Iranians with heart failure. Eval. Health Prof..

[23] Pakpour A.H., Lawson D.M., Tadakamadla S.K., Fridlund B. (2016). Validation of Persian rapid estimate of adult literacy in dentistry. J. Investig. Clin. Dent..

[24] Marufkhani V., Mohammadi F., Mirzadeh M., Allen K.A., Motalebi S.A. (2021). Leisure activity engagement as a predictor for quality of life in Community-Dwelling older adults. Asian J. Soc. Health Behav..

[25] Yahaghi R., Ahmadizade S., Fotuhi R., Taherkhani E., Ranjbaran M., Buchali Z., Jafari R., Zamani N., Shahbazkhania A., Simiari H. (2021). Fear of COVID-19 and perceived COVID-19 infectability supplement theory of planned behavior to explain Iranians’ intention to get COVID-19 vaccinated. Vaccines.

[26] Nejati B., Fan C.-W., Broome W.J., Lin C.-Y., Griffiths M.D., Pakpouir A.H. (2021). Validating the Persian Intuitive Eating Scale-2 among breast cancer survivors who are overweight or obese. Eval. Health Prof..

[27] Poon L.Y.J., Tsang H.W.H., Chan T.Y.J., Man S.W.T., Ng L.Y., Wong Y.L.E., Lin C.-Y., Chien C.-W., Griffiths M.D., Pontes H.M. (2021). Psychometric properties of the Internet Gaming Disorder Scale–Short-Form (IGDS9-SF): A systematic review. J. Med. Internet Res..

[28] Lin C.-Y., Tsai C.-S., Jian C.-R., Chao S.-R., Wang P.-W., Lin H.-C., Huang M.-F., Yeh Y.-C., Liu T.-L., Chen C.-S. (2022). Comparing Three Versions of UCLA Loneliness Scale (Full, Eight-Item, and Three-Item Versions) in Individuals with Schizophrenia and Schizoaffective Disorder: Psychometric Properties Evaluation. Int. J. Environ. Res. Public Health.

[29] Peterson R., Kim Y., Choi B. (2020). A Meta-Analysis of Construct Reliability Indices and Measurement Model Fit Metrics. Methodology.

[30] Mamun M.A., Alimoradi Z., Gozal D., Manzar M.D., Broström A., Lin C.-Y., Huang R.-Y., Pakpour A.H. (2021). Validating Insomnia Severity Index (ISI) in a Bangladeshi Population: Using Classical Test Theory and Rasch Analysis. Int. J. Environ. Res. Public Health.

[31] Cameron I.M., Scott N.W., Adler M., Reid I.C. (2014). A comparison of three methods of assessing differential item functioning (DIF) in the Hospital Anxiety Depression Scale: Ordinal logistic regression, Rasch analysis and the Mantel chi-square procedure. Qual. Life Res..

[32] Fan C.-W., Chang K.-C., Lee K.-Y., Yang W.-C., Pakpour A.H., Potenza M.N., Lin C.-Y. (2022). Rasch Modeling and Differential Item Functioning of the Self-Stigma Scale-Short Version Among People with three different Psychiatric Disorders. Int. J. Environ. Res. Public Health.

[33] Chang K.-C., Wang J.-D., Tang H.-P., Cheng C.-M., Lin C.-Y. (2014). Psychometric evaluation using Rasch analysis of the WHOQOL-BREF in heroin-dependent people undergoing methadone maintenance treatment: Further item validation. Health Qual. Life Outcomes.

[34] Lin C.-Y., Imani V., Griffiths M.D., Pakpour A.H. (2021). Psychometric properties of the Persian Generalized Trust Scale: Confirmatory factor analysis and Rasch models and relationship with quality of life, happiness, and depression. Int. J. Ment. Health Addiction.

[35] Ratzan S.C., Parker R.M., Selden C.R., Zorn M., Ratzan S.C., Parker R.M. (2000). Introduction. National Library of Medicine Current Bibliographies in Medicine: Health Literacy.

