# Correction: Alijanzadeh et al. Measurement Invariance and Differential Item Functioning of the Health Literacy Instrument for Adults (HELIA): A Large-Scale Cross-Sectional Study in Iran. *Healthcare* 2022, *10*, 2064

**DOI:** 10.3390/healthcare10122541

**Published:** 2022-12-15

**Authors:** Mehran Alijanzadeh, Chung-Ying Lin, Rafat Yahaghi, Jalal Rahmani, Nahid Yazdi, Elahe Jafari, Hashem Alijani, Narges Zamani, Razie Fotuhi, Elham Taherkhani, Zeinab Buchali, Robabe Jafari, Narges Mahmoudi, Leila Poorzolfaghar, Safie Ahmadizade, Azam Shahbazkhania, Zainab Alimoradi, Amir H. Pakpour

**Affiliations:** 1 Social Determinants of Health Research Center, Research Institute for Prevention of Non-Communicable Diseases, Qazvin University of Medical Sciences, Qazvin 3419759811, Iran; mehranalijanzadeh@gmail.com (M.A.); yahaghirafat@yahoo.com (R.Y.); jrahmani49@gmail.com (J.R.); n.yazdi@qums.ac.ir (N.Y.); elhejafari@gmail.com (E.J.); hs.alijani@gmail.com (H.A.); zamani.ee@gmail.com (N.Z.); m.r.a.mafi@gmail.com (R.F.); etaherkhani.mid1@gmail.com (E.T.); bochalizeinab@gmail.com (Z.B.); ro.midewife@yahoo.com (R.J.); mahmoudi.narges@yahoo.com (N.M.); poorzolfaghar6565@gmail.com (L.P.); ahmadizadehn@yahoo.com (S.A.); aazam.shahbazkhani@gmali.com (A.S.); zainabalimoradi@yahoo.com (Z.A.); 2 Institute of Allied Health Sciences, College of Medicine, National Cheng Kung University, Tainan 70101, Taiwan; cylin36933@gmail.com; 3 Biostatistics Consulting Center, National Cheng Kung University Hospital, College of Medicine, National Cheng Kung University, Tainan 70101, Taiwan; 4 Department of Nursing, School of Health and Welfare, Jönköping University, 553 18 Jönköping, Sweden

There were some errors in the original publication in the Abstract and Results sections [1]. *Abstract:* (N = 9678; 67.3% females; mean age = 36.44 years); *Results*: 9678 participants (3073 males; 31.4%).

A correction has been made to *Abstract*, *line 6*: **(N = 9775; 67.3% females; mean age = 36.44 years)**; *Results*, *Paragraph 1*, *line 1*: **9775 participants (3073 males; 31.4%)**.

Table 1 has also been corrected with respect to the total number of participants as well as adding missing values to gender, city, educational status, accommodation and marital status. The DIF contrast across the age groups was then corrected in Section 3 and Table 2.

The authors state that the scientific conclusions are unaffected. This correction was approved by the Academic Editor. The original publication has also been updated.

## Figures and Tables

**Table 1:** Participant characteristics (*N* = 9775).

	Mean ± SD or *n* (%)
**Age (years)**	36.44 ± 11.97
**Gender**	
Male	3073 (31.4)
Female	6576 (67.3)
Missing	126 (1.3)
**City**	
Qazvin	4655 (47.6)
Takestan	1216 (12.4)
Avaj	330 (3.4)
Abyek	657 (6.7)
Bueenzahra	963 (9.9)
Alborz	1867 (19.1)
Missing	87 (0.9)
**Educational status**	
Primary school	1082 (11.1)
Secondary school	1576 (16.1)
Diploma	3220 (32.9)
University	3851 (39.4)
Missing	46 (0.5)
**Accommodation**	
City	7287 (74.6)
Rural	2309 (23.6)
Missing	179 (1.8)
**Marital status**	
Single	1779 (18.2)
Married	6987 (71.5)
Divorced/widowed	90 (0.9)
Missing	919 (9.4)

**Table 2:** Psychometric properties of the Health Literacy Instrument for Adults at the item level.

Item #	Analyses from Classical Test Theory				Rasch Analyses							
	Factor Loading *^,†^	Item-Total Correlation	S	K	Infit MnSq	Outfit MnSq	Difficulty	Discrimination	DIF Contrast across Gender ^§,¶^	DIF Contrast across Education ^§,#^	DIF Contrast across Accommodation ^§,#^	DIF Contrast across Age ^§,#^
Re-1	0.795	0.724	0.795	0.375	1.16	1.18	−0.38	0. 79	0.10	−0.23	0.11	0.00
Re-2	0.846	0.788	0.846	0.106	0.89	0.89	0.05	1.12	−0.22	0.00	−0.05	0.16
Re-3	0.903	0.816	0.903	0.079	0.81	0.80	0.04	1.21	0.00	0.08	0.10	−0.18
Re-4	0.921	0.753	0.921	−0.110	1.10	1.10	0.29	0.91	−0.10	0.17	−0.15	0.00
AC-1	0.833	0.780	0.833	−0.058	1.00	1.03	−0.01	0.97	0.13	0.16	0.05	−0.14
AC-2	0.850	0.803	0.850	0.096	0.88	0.86	−0.31	1.13	−0.10	−0.14	0.15	−0.18
AC-3	0.886	0.810	0.886	−0.441	0.91	0.92	0.50	1.09	−0.04	−0.11	0.00	−0.07
AC-4	0.879	0.824	0.879	−0.167	0.83	0.82	0.13	1.19	−0.09	−0.08	0.00	−0.09
AC-5	0.859	0.794	0.859	−0.134	0.99	0.99	0.20	1.02	−0.10	0.02	−0.10	0.36
AC-6	0.822	0.723	0.822	0.323	1.36	1.32	−0.51	0.65	0.23	0.16	−0.15	0.12
Un-1	0.700	0.719	0.700	1.283	1.05	1.11	−0.31	0.91	−0.07	−0.16	0.06	0.06
Un-2	0.688	0.758	0.688	1.423	0.93	0.90	−0.40	1.10	−0.10	−0.10	0.04	0.05
Und-3	0.811	0.760	0.811	0.373	0.98	0.99	0.36	1.03	0.10	0.14	−0.11	0.00
Un-4	0.770	0.795	0.770	1.060	0.83	0.84	−0.05	1.18	0.19	0.11	0.00	−0.05
Un-5	0.706	0.768	0.706	1.804	0.96	0.90	−0.52	1.09	−0.07	0.00	0.15	−0.13
Un-6	0.774	0.759	0.774	0.547	0.93	0.97	0.17	1.05	−0.02	−0.28	0.08	0.00
Un-7	0.839	0.694	0.839	−0.003	1.28	1.30	0.76	0.70	0.00	0.24	−0.20	0.04
Ap-1	0.906	0.726	0.906	−0.177	1.11	1.09	0.33	0.92	0.00	0.56	−0.15	−0.35
Ap-2	0.847	0.782	0.847	−0.059	0.81	0.80	0.17	1.21	0.10	−0.10	0.00	0.20
Ap-3	0.810	0.777	0.810	0.009	0.81	0.82	0.00	1.18	0.06	−0.23	0.04	0.12
Ap-4	0.782	0.666	0.782	0.424	1.22	1.25	−0.50	0.72	−0.18	−0.26	0.04	0.05
De-1	0.766	0.644	0.766	0.327	0.94	1.06	−0.25	0.98	−0.07	0.17	0.08	−0.35
De-2	0.689	0.660	0.689	1.007	0.97	0.97	−0.62	1.04	−0.20	0.10	0.00	0.00
De-3	0.714	0.662	0.714	0.084	1.04	1.11	−0.12	0.96	−0.08	−0.05	0.00	0.05
De-4	0.774	0.632	0.774	−0.429	1.30	1.31	0.16	0.72	0.04	0.00	0.00	−0.06
De-5	0.743	0.741	0.743	−0.110	0.77	0.79	−0.03	1.25	−0.07	−0.12	0.09	0.07
De-6	0.766	0.631	0.766	−0.969	1.26	1.29	0.79	0.68	0.13	−0.03	−0.18	0.13
De-7	0.732	0.732	0.732	0.019	0.74	0.75	−0.12	1.26	0.07	−0.12	0.14	−0.12
De-8	0.757	0.717	0.757	0.142	0.82	0.81	−0.23	1.20	−0.13	−0.10	0.14	−0.13
De-9	0.787	0.694	0.787	−0.294	0.99	1.0	0.22	1.03	0.00	0.12	−0.16	0.00
De-10	0.731	0.696	0.731	−0.349	0.92	0.97	0.33	1.06	0.00	−0.10	0.04	0.06
De-11	0.682	0.620	0.682	0.403	1.21	1.24	−0.32	0.81	0.22	0.22	−0.19	0.28
De-12	0.786	0.710	0.786	−0.244	0.94	0.96	0.18	1.08	0.00	−0.03	0.03	0.00

* All factor loadings were significant at 0.001; S = skewness; K = kurtosis; Re = reading; Ac = access to information; Un = understanding; Ap = appraisal; De = decision making/behavioral intention. ^†^ Based on first-order confirmatory factor analysis (CFA). CFA model fit: *χ*^2^ (*df*) = 5052.419 (485); comparative fit index = 0.993; Tucker-Lewis index = 0.993; root mean square error of approximation (90% CI) = 0.033 (0.032,0.034); and standardized root mean square residual = 0.041. ^§^ DIF contrast >1 indicates substantial DIF. ^¶^ DIF contrast across gender = difficulty for females − difficulty for males. ^#^ DIF contrast across education = difficulty for participants with lower education (lower than diploma) − difficulty for participants with higher education (higher than diploma). ^#^ DIF contrast across accommodation = difficulty for participants living in city areas − difficulty for participants living in rural areas. ^#^ DIF contrast across age = difficulty for younger-aged participants (<36.44 years) − difficulty for older-aged participants (≥36.44 years). MnSq = mean square error; DIF = differential item functioning; S = skewness; K = kurtosis.

## References

[1] Alijanzadeh M., Lin C.-Y., Yahaghi R., Rahmani J., Yazdi N., Jafari E., Alijani H., Zamani N., Fotuhi R., Taherkhani E. (2022). Measurement Invariance and Differential Item Functioning of the Health Literacy Instrument for Adults (HELIA): A Large-Scale Cross-Sectional Study in Iran. Healthcare.

